# The Aim and Scope of Women's Work in Relation to Public Health

**Published:** 1907-05-11

**Authors:** 


### THE AIM AND SCOPE OF WOMEN'S WORK IN RELATION TO PUBLIC HEALTH.

In a recent discussion at the Royal Sanitary Institute, introduced by Dr. Meredith Richards, the Medical Officer of Health for Croydon, the problem of the aptitude of woman for work in connection with public health did not escape consideration. Man is naturally conservative, and it is certain that every fresh enlargement of woman's sphere of activity will have to be justified in the face of the primordial hostility which it is the fate of novelty, and especially of feminine novelty, to have arraigned against it.

Nevertheless, there will be few who have considered the problem of sex in relation to aptitudes who will differ from Dr. Richards in his insistence on the differences, rather than the similarities of aptitude in the sexes. "It seems," said Dr. Richards, "to be too often forgotten that there are fundamental differences, not only in the training, but in both the physical and mental capabilities of men and women, and that due regard should be paid to these differences in allotting to men and women respectively their true place in public health work. A firm in the city, which had on its staff a good linguist and an able accountant, would show little common sense if they selected the former as their book-keeper and relegated their foreign correspondence to the man of figures. Similarly, in public health work, men and women have different aptitudes, and we are not making the best of our human material unless we recognise these aptitudes, and assign to each the task which they can do most efficiently and, therefore with greatest ease to themselves and advantage to the community.

"To avoid misconception, it may be well to insist that this is not a question of superiority or inferiority; it is simply a protest against ill-advised attempts to put square pegs into round holes. In fact, it is just because women can do certain things better than men can, that they should be selected for those posts in which their special capabilities can have free play, and it is equally because they do other things less well that they should not be given posts for which they are by nature unsuited."

Dr. Richards then summarises the work that can be most profitably discharged by women as follows:—

(*a*) Visiting newly-born infants.

(*b*) Visiting the poorer houses and giving practical instruction in personal hygiene.

(*c*) Supervising cases of phthisis.

(*d*) Supervising the practice and inspecting the homes of midwives.

(*e*) Giving popular lectures on personal hygiene and on the management of infants.

(*f*) Inspecting under the Infant Life Protection Act.

(*g*) Management of milk depôts.

(*h*) Inspection of workshops where women are employed, and supervision of out-workers.

(*i*) Supervision of houses let in lodgings.

(*k*) Visiting cases of measles, whooping-cough, ringworm, etc., reported from the public elementary schools, and securing treatment for these diseases.

(*l*) Visiting schools for the detection of verminous conditions and ringworm.

In all this we agree with Dr. Richards. It is one of the excusable mistakes of women that, in entering a new field of work, they have wished to do so as the "equals" of men. While excusable, it is a profound error. Women are not the equals of men, or, if they prefer it, men are not the equals of women, and the success of women in public health work will depend upon a recognition of this inequality. Undoubtedly there is an important work for women in public health administration, but this work is not that of the sanitary inspector; and it is a mistake to confuse the different character of the work the two sexes can respectively perform by the same descriptive title. "Health visitor," the name by which many of these workers are now known, seems in the minds of some to designate inferiority. To our mind, the title is appropriate, and is far from connoting an inferior position to that of sanitary inspector; it is, indeed, distinctive of a class of work which in many respects is of a higher order. Supplementing as she does the work of the practitioner in educating mothers in the rudiments of domestic hygiene, she combines the knowledge of the inspector of nuisances with the trained intelligence of a skilled nurse, and in this there is scope for the exercise of her prerogative of tact in a degree far above what is called for in statutory administration. That her status should be appropriately recognised by the sanitary authorities for whom she works goes without saying, but her privileges will necessarily be of a different order from those of the sanitary inspector. The very nature of the work which it is most urgently hers to do, forbids a display of statutory powers such as is unfortunately essential to the sanitary supervision of premises. The most urgent sanitary reform of to-day is the domestic reform of woman herself, and by women with women's ways must this be done.

